# High Nasopharyngeal Carriage of Non-Vaccine Serotypes in Western Australian Aboriginal People Following 10 Years of Pneumococcal Conjugate Vaccination

**DOI:** 10.1371/journal.pone.0082280

**Published:** 2013-12-03

**Authors:** Deirdre A. Collins, Anke Hoskins, Jacinta Bowman, Jade Jones, Natalie A. Stemberger, Peter C. Richmond, Amanda J. Leach, Deborah Lehmann

**Affiliations:** 1 Telethon Institute for Child Health Research, Centre for Child Health Research, the University of Western Australia, Perth, Western Australia, Australia; 2 Division of Microbiology & Infectious Diseases, PathWest Laboratory Medicine WA, Perth, Western Australia, Australia; 3 Microbiology & Immunology, School of Pathology and Laboratory Medicine, the University of Western Australia, Perth, Western Australia, Australia; 4 School of Paediatrics and Child Health, University of Western Australia, Perth, Western Australia, Australia; 5 Menzies School of Health Research, Charles Darwin University, Darwin, Northern Territory, Australia; Instituto Butantan, Brazil

## Abstract

**Background:**

Invasive pneumococcal disease (IPD) continues to occur at high rates among Australian Aboriginal people. The seven-valent pneumococcal conjugate vaccine (7vPCV) was given in a 2-4-6-month schedule from 2001, with a 23-valent pneumococcal polysaccharide vaccine (23vPPV) booster at 18 months, and replaced with 13vPCV in July 2011. Since carriage surveillance can supplement IPD surveillance, we have monitored pneumococcal carriage in western Australia (WA) since 2008 to assess the impact of the 10-year 7vPCV program.

**Methods:**

We collected 1,500 nasopharyngeal specimens from Aboriginal people living in varied regions of WA from August 2008 until June 2011. Specimens were cultured on selective media. Pneumococcal isolates were serotyped by the quellung reaction.

**Results:**

*Streptococcus pneumoniae*, *Haemophilus influenzae* and *Moraxella catarrhalis* were carried by 71.9%, 63.2% and 63.3% respectively of children <5 years of age, and 34.6%, 22.4% and 27.2% of people ≥5 years. Of 43 pneumococcal serotypes identified, the most common were 19A, 16F and 6C in children <5 years, and 15B, 34 and 22F in older people. 7vPCV serotypes accounted for 14.5% of all serotypeable isolates, 13vPCV for 32.4% and 23vPPV for 49.9%, with little variation across all age groups. Serotypes 1 and 12F were rarely identified, despite causing recent IPD outbreaks in WA. Complete penicillin resistance (MIC ≥2µg/ml) was found in 1.6% of serotype 19A (5.2%), 19F (4.9%) and 16F (3.2%) isolates and reduced penicillin susceptibility (MIC ≥0.125µg/ml) in 24.9% of isolates, particularly 19F (92.7%), 19A (41.3%), 16F (29.0%). Multi-resistance to cotrimoxazole, tetracycline and erythromycin was found in 83.0% of 23F isolates. Among non-serotypeable isolates 76.0% had reduced susceptibility and 4.0% showed complete resistance to penicillin.

**Conclusions:**

Ten years after introduction of 7vPCV for Aboriginal Australian children, 7vPCV serotypes account for a small proportion of carried pneumococci. A large proportion of circulating serotypes are not covered by any currently licensed vaccine.

## Introduction

Invasive pneumococcal disease (IPD), which includes pneumonia, meningitis, and septicaemia, causes an estimated 476,000 deaths in children < 5 years annually, the majority of which occur in the third world [1]. In Western Australia (WA) between 1997 and 2007, the overall IPD incidence rate was 47 cases per 100,000 population per year in Aboriginal people, 6.7 times higher than the IPD incidence in non-Aboriginal people [2]. Furthermore, Aboriginal children experience high rates of otitis media (OM), often caused by *Streptococcus pneumoniae*, with up to 21% of children in WA having a tympanic membrane perforation before the age of 2 years in remote areas [3]. OM and its complications can have profound effects on hearing and subsequently can impact on speech and language development and behaviour. Overcrowding and indoor smoking are common in the Aboriginal population and associated with increased risk of nasopharyngeal bacterial carriage which is a necessary precursor to IPD and OM [4].

In 2001 the seven-valent pneumococcal conjugate vaccine (7vPCV, Prevenar^®^), covering serotypes 4, 6B, 9V, 14, 18C, 19F and 23F, was introduced for Aboriginal Australian children in a 2-4-6-month schedule, with a catch-up schedule for children < 2 years of age, and for children < 5 years of age with predisposing medical conditions. A booster of 23-valent pneumococcal polysaccharide vaccine (23vPPV, Pneumovax 23^®^, covers 7vPCV serotypes and 1, 2, 3, 5, 7F, 8, 9N, 10A, 11A, 12F, 15B, 17F, 19A, 20, 22F and 23F) was offered at age 18 months to Aboriginal children. In 2005 the 7vPCV program was extended to include all Australian children. On 1 July 2011 13vPCV replaced 7vPCV (covering six additional serotypes; 1, 3, 5, 6A, 7F, 19A) in the immunization schedule and a fourth dose of 13vPCV replaced the 23vPPV booster in Aboriginal children. Since the introduction of 7vPCV, the incidence of IPD caused by vaccine serotypes has fallen but IPD due to non-7vPCV serotypes has increased, and almost doubled in WA Aboriginal adults aged 30-49 years [2]. Serotypes which have emerged since the introduction of 7vPCV in WA include 1, 12F and 19A [5].

Surveillance of IPD in WA is limited by the need to administer antibiotics in remote areas before a sample of blood for culture can be collected at a referral hospital, and by the low sensitivity of blood culture. In addition, the Aboriginal population in WA is relatively small (around 77,000), and while the incidence of IPD is high, small numbers of cases make it difficult to monitor trends effectively. Given these limitations, surveillance of nasopharyngeal pneumococcal carriage assists in identifying serotypes circulating in the population, helping to predict changes in serotypes causing IPD [6]. Carriage studies facilitate monitoring the impact of PCV programs, which alter carriage and associated herd immunity. Studying pneumococcal carriage also allows antimicrobial susceptibility patterns to be monitored which can guide management of common non-invasive infections such as OM and pneumonia.

While few reports exist on pneumococcal carriage in Aboriginal Australians prior to the introduction of 7vPCV, carriage studies in the Northern Territory indicate that 7vPCV reduced carriage of 7vPCV serotypes, similar to trends observed elsewhere [7–9].

Since 2008, we have studied nasopharyngeal carriage of bacterial pathogens in the Aboriginal population in WA. Our aim is to monitor nasopharyngeal carriage of pneumococcal serotypes, as well as the prevalence of other commonly carried pathogens, namely *Haemophilus influenzae*, *Moraxella catarrhalis* and *Staphylococcus aureus*, in Aboriginal children and adults living in Western Australia. Here we report on carriage rates of bacteria, and serotype distribution and antimicrobial susceptibility of pneumococci in children and adults in WA until the introduction of 13vPCV.

## Methods

### Setting

**Figure 1:**
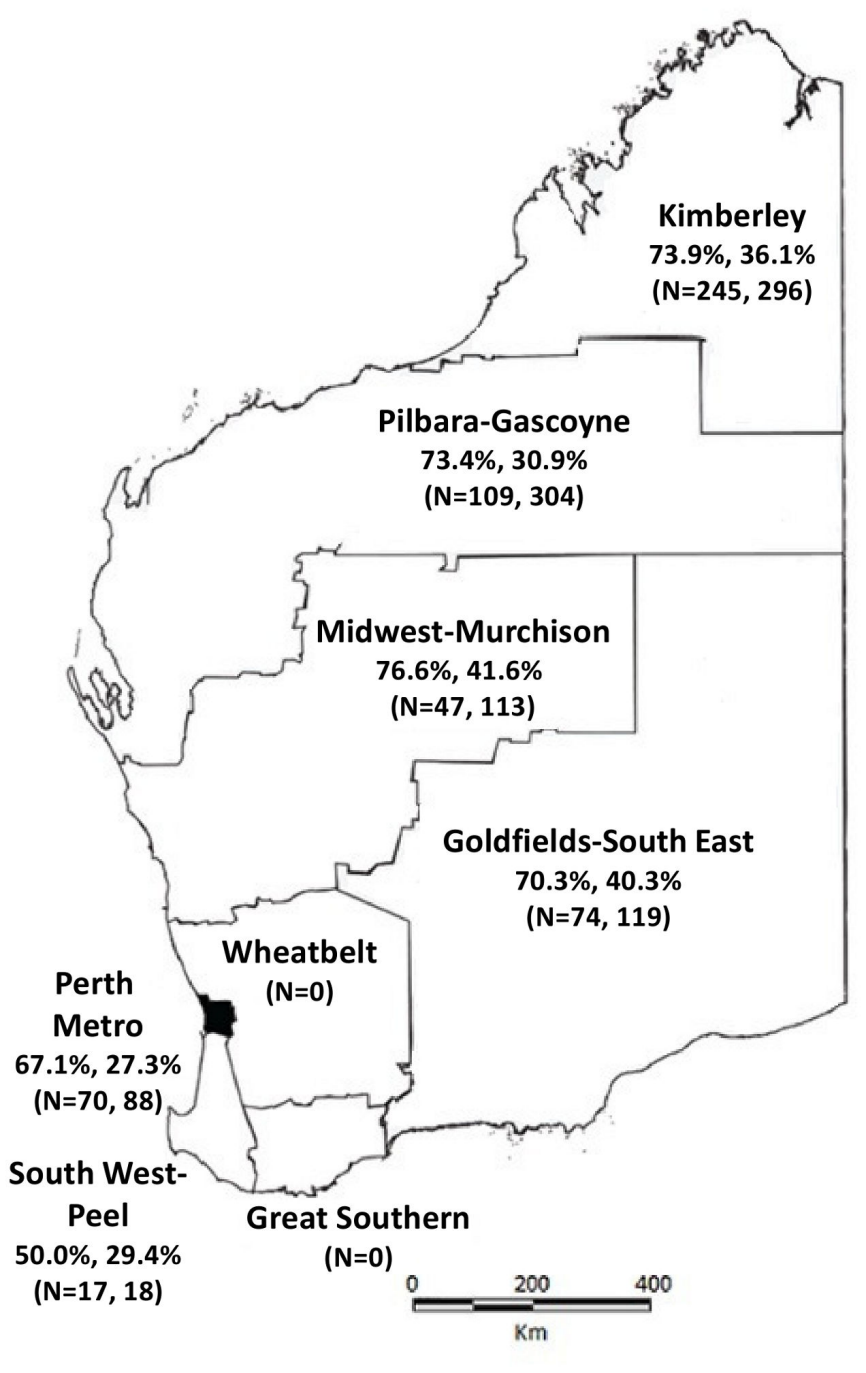
Prevalence of *S. pneumoniae* carriage in 1500 Aboriginal people (<5 years, ≥ 5 years) by Western Australian health regions. N = number of nasopharyngeal swabs collected per region for participants (< 5 years, ≥ 5 years). Overall, the prevalence of *S*. *pneumoniae* carriage was 71.9% in children < 5 years, and 34.6% in people ≥ 5 years. Specimens were collected 1 Aug 2008 - 30 June 2011.

WA covers an area of 2.5 million km^2^, with a range of climates from tropical northern Kimberley to the warm temperate southwest coast, to the inland desert. There is a sparse population of 2.2 million people (population density 0.9 persons/km^2^) (Figure 1). Aboriginal people make up 3% of the population. Approximately two-thirds live in non-metropolitan regions, in communities ranging from small and very remote (e.g. Laverton; Aboriginal population 339 among 1,227 in total) to regional towns (e.g. Kalgoorlie; 889 among 13,949 total) [10]. In the Perth metropolitan region, Aboriginal people numbered 12,852 among 1.7 million in the 2011 Census [10]. 

### Study Method

#### Procedures

From August 2008 until June 2011, we visited communities with Aboriginal populations across WA for data collection. Study participants attending health services for routine examination, immunization or illness who identified as Aboriginal were recruited opportunistically. People were also recruited during home visits. Non-Aboriginal people were excluded, as were those with severe congenital abnormalities.

After obtaining informed written consent, demographic and environmental data were collected including smoking behaviour of family members and the number of people sharing a home. We recorded information about past and present health status and recent antibiotic use. Where possible, medical records were examined to obtain further details on recently prescribed antibiotics and illness. The Australian Childhood Immunisation Register (ACIR) was later consulted to record the vaccination status of children born from 1998 onwards. ACIR records were accessed using participants’ names and dates of birth. Children were classified as “vaccinated” with 7vPCV if they had received at least two doses of 7vPCV at least 2 weeks prior to specimen collection.

Nasopharyngeal swabs (NPS) were collected using a nylon flocked swab (Copan Diagnostics Inc., USA). If participants were reluctant to provide a NPS, a nose-blown sample on a clean tissue was swabbed [11]. Swabs were stored in 1 mL skim milk-tryptone-glucose-glycerol broth in a portable cooler until transfer to a liquid nitrogen dry shipper at ≤ -80°C within 12 hours for transport by road or air to PathWest Laboratory Medicine WA in Perth, where they were stored at -80°C.

#### Microbiological methods

Primary culture was carried out as described previously [12]. Briefly, swabs were cultured on selective media for common nasopharyngeal bacteria. Subcultured isolates were confirmed as *S. pneumoniae* by optochin susceptibility, and *H. influenzae, M. catarrhalis* and *S. aureus* were confirmed by standard criteria [12]. Two pneumococcal isolates (or more, if morphologically distinct) per positive culture were stored and serotyped by the Quellung reaction using antisera obtained from the Statens Serum Institut, Denmark. Serotypes were validated at Menzies School of Health Research, Darwin, if an inconclusive Quellung result was obtained. Isolates that could not be serotyped by Quellung are referred to as “non-serotypeable”. Susceptibility to penicillin, ceftriaxone, cotrimoxazole, erythromycin, chloramphenicol and tetracycline was determined using the disc diffusion method. Minimum inhibitory concentrations (MICs) to penicillin and ceftriaxone in isolates showing reduced susceptibility by disc diffusion were determined by E-test (bioMérieux Diagnostics, France). Antibiotic resistance was classified according to the Clinical and Laboratory Standards Institute guidelines [13].

#### Analysis

Since a minimum of two isolates were serotyped for each sample, results were aggregated according to serotype to calculate the proportion of individual serotypes among all isolates. Descriptive analyses were performed using SPSS 15.0 for Windows. Proportions were compared between different groups using the χ^2^ test.

### Ethics approval and community consultation

Ethical approval to conduct this study was granted by the Princess Margaret Hospital for Children Ethics Committee, the Western Australian Country Health Service Board Research Ethics Committee and the Western Australian Aboriginal Health Ethics Committee. Approval to approach communities in the Kimberley was also granted by the Kimberley Aboriginal Health Planning Forum. Prior to planning a visit, we consulted local Aboriginal community committees and/or councils to inform them of our study and seek approval to visit at an appropriate time. Written informed consent was obtained from next of kin, caretakers or guardians on behalf of children participants involved in the study, and adults provided written consent for their own participation.

## Results

### Study population

We visited 40 communities across WA in the following regions: the Kimberley, Goldfields, Pilbara, Gascoyne, as well as the metropolitan area of Perth and its surroundings (Figure 1). From August 2008 to June 2011, 1,473 NPS and 27 nose-blown samples were collected and 1,430 questionnaires were completed. Participants ranged in age from < 24 hours to 101 years. While 44.9% of all participants were male, among adults there were more female than male participants (Table 1). The number of participants aged < 5 years or ≥ 5 years by region is shown in Figure 1. At the time of specimen collection, 179/1430 (12.5%) people reported they were sick, 107 (7.5%) reported respiratory symptoms. Eight hundred and seven (56.4%) lived in a home where at least one person smoked inside, and 880 (61.5%) lived in a home with at least six inhabitants. Sixty-eight (4.8%) reported taking antibiotics in the previous 4 weeks, 17 of whom were still taking them at the time of swab collection. Of 861 children born from 1998 onwards, 486 (56.4%) could be identified on ACIR, and 464 (95.4%) of these had received at least two doses of 7vPCV prior to specimen collection.

**Table 1:** Number of swabs collected, proportion collected from male participants (%) by age group and age-specific prevalence (%) of *S. pneumoniae*, *H. influenzae*, *M. catarrhalis* and *S. aureus* carriage.

**Age**	**Number of swabs**	**Male**	***S. pneumoniae***	***H. influenzae***	***M. catarrhalis***	***S. pneumoniae*, *H. influenzae* and *M. catarrhalis***	***S. aureus***
< 6 mth	73	53.4	49.3	41.1	47.9	17.8	30.1
6-11 mth	61	59.0	85.2	65.6	60.6	34.5	11.5
12-23 mth	126	61.1	76.9	71.4	66.7	49.0	7.1
2-4 yr	302	51.3	72.5	64.6	66.2	44.5	8.3
5-14 yr	476	52.3	49.4	35.7	41.2	18.0	16.1
15-29 yr	218	22.5	20.6	9.0	11.0	3.2	13.8
30-49 yr	171	26.9	20.5	10.5	12.3	3.5	7.0
50-64 yr	52	25.0	11.5	1.9	15.4	0	7.7
≥ 65 yr	21	42.9	19.0	4.7	28.6	0	14.3
**Total**	**1500**	**44.9**	**48.6**	**37.7**	**40.7**	**21.9**	**12.6**

### Carriage rates by age group

*S. pneumoniae*, *H. influenzae* and *M. catarrhalis* were carried by 71.9%, 63.2% and 63.3% respectively of children < 5 years of age, and 34.6%, 22.4% and 27.2% of people ≥ 5 years. Pneumococcal carriage rates were 74.1% in females and 70.0% in males < 5 years, and 30.6% in females and 40.9% in males ≥ 5 years. More detailed age-specific bacterial carriage rates are shown in Table 1. Among infants aged < 6 months, 49.3% carried *S. pneumoniae*. The highest rate of pneumococcal carriage was in children aged 6-11 months (Table 1). Of particular note, 33.3% of infants < 2 months of age, who were not yet eligible for their first dose of 7vPCV, carried *S. pneumoniae* (not shown). We found no significant difference in pneumococcal carriage in people reporting respiratory symptoms compared to those who were not (p = 0.24).

Region-specific *S. pneumoniae* carriage rates for participants < 5 and ≥ 5 years are shown in Figure 1. No swabs were collected in the Wheatbelt and Great Southern regions of WA. In children < 5 years carriage rates ranged from 50.0% in the South West-Peel region to 76.6% in the Midwest-Murchison region; in older people carriage rates were highest in the Midwest-Murchison region (41.6%) and lowest in the Perth Metro area (27.3%, Figure 1). 

Simultaneous carriage of *S. pneumoniae*, *H. influenzae* and *M. catarrhalis* was observed in 40.9 % of children < 5 years. Among adults, *M. catarrhalis* was most commonly carried in those aged ≥ 50 years, while *H. influenzae* was rarely carried in adults ≥ 50 years (Table 1). The carriage rate of *S. aureus* was 12.6%, the highest rate being in children aged < 6 months (30.1%, Table 1). Other bacteria that were carried less frequently included Lancefield Group A streptococci (1.6% overall) and Lancefield Group C streptococci (0.9%).

### Serotype distributions

**Figure 2:**
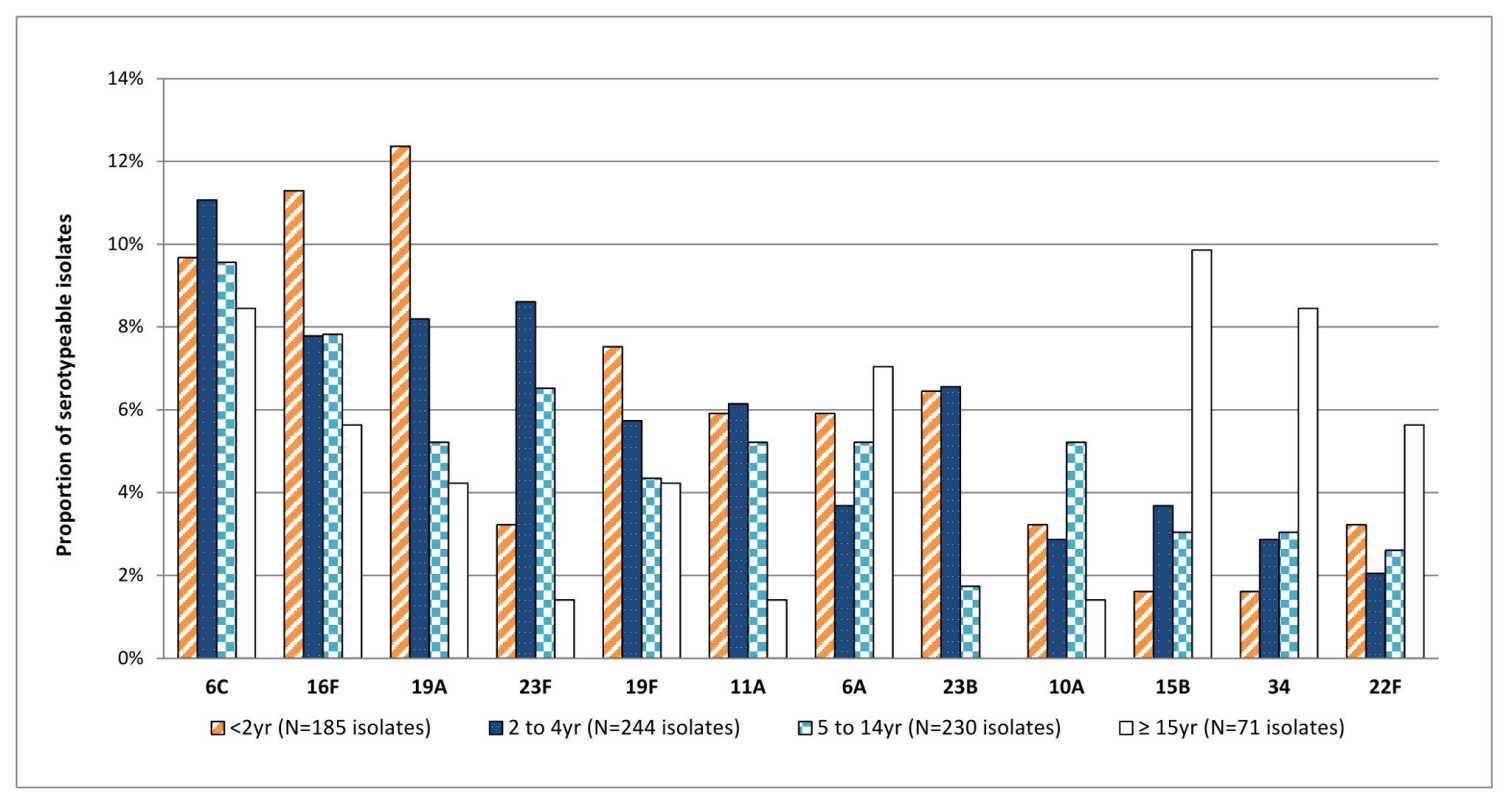
Distribution of the 12 most common pneumococcal serotypes by age group.

Among 729 pneumococcus-positive cultures we identified 856 distinct isolates, of which 730 were typed to 43 different serotypes, and 126 (14.7%) were non-serotypeable (Table 2). The age-specific distribution of the 12 most common serotypes is shown in Figure 2. For children < 2 years, the most common carriage serotypes were 19A, 16F, 6C, and 19F. In children aged 2-14 years, the most common serotypes were 6C, 23F, 19A and 16F. The most common serotypes in adults were 15B, 34 and 6C. Overall, 114 (15.6%) pneumococcus carriers of all ages had two strains identified, while six carriers had three strains identified. One strain was nonserotypeable in 55 (45.8%) cases of multiple strain carriage and the most common serotype was 16F in 17 (13.8%) cases. 

**Table 2:** Frequencies of serotypes and antibiotic resistance rates found among 856 isolates of *S. pneumoniae*.

**Serotype**	**Number of isolates**	**cotr** ^R^ (%)	**ery** ^R^ (%)	**tet** ^R^ (%)	**pen** ^I^ (%)	**pen** ^R^ (%)
1	7	0	0	0	0	0
3	17	0	0	0	0	0
4	5	0	0	0	0	0
6A	37	8.1	62.1	0	2.7	0
6B	3	0	33.3	0	0	0
6C	73	73.9	4.1	0	0	0
7C	12	0	16.6	0	0	0
7F	12	0	0	0	0	0
8	6	0	0	0	0	0
9A	1	0	0	0	0	0
9N	8	12.5	87.5	87.5	0	0
9V	11	100.0	0	0	100.0	0
10A	26	80.7	0	0	0	0
10F	5	100.0	0	0	0	0
11A	39	15.9	0	0	5.1	0
12F	7	14.3	0	0	0	0
13	1	0	0	0	0	0
15A	6	50.0	100.0	50.0	50.0	0
15B	26	7.7	11.5	0	15.4	0
15C	16	25.0	0	0	0	0
16F	62	4.8	9.7	8.1	29.0	3.2
17F	9	0	44.4	0	11.1	0
18A	7	0	0	0	0	0
18C	3	100.0	0	0	0	0
19A	58	48.3	10.3	31.0	41.3	5.2
19F	41	2.4	0	0	92.7	4.9
20	2	0	0	0	0	0
21	8	37.5	0	0	0	0
22A	14	14.3	0	0	0	7.1
22F	21	0	71.4	0	0	0
23A	13	0	0	0	0	0
23B	32	25.0	0	0	21.9	0
23F	43	90.7	90.7	86.0	4.7	2.3
29	1	0	0	0	0	0
31	12	0	16.6	0	0	0
33B	4	0	50.0	0	50.0	0
33D	2	0	100.0	0	100.0	0
33F	21	19.0	4.8	0	0	0
34	23	0	0	0	0	0
35B	15	6.7	0	0	13.3	0
35F	10	10.0	0	0	0	0
37	1	0	0	0	0	0
38	10	0	0	0	0	0
NT	126	46.9	27.0	9.5	76.2	4.0
**TOTAL**	**856**	**30.7**	**18.2**	**9.5**	**24.9**	**1.6**

43 serotypes were identified among 730 serotypeable isolates, 126 isolates were non-serotypeable (NT). cotr: cotrimoxazole, ery: erythromycin, tet: tetracycline, pen: penicillin, ^R^: resistant, ^I^: intermediate resistance

### Proportion of pneumococcal carriage serotypes covered by different vaccine formulations

**Figure 3:**
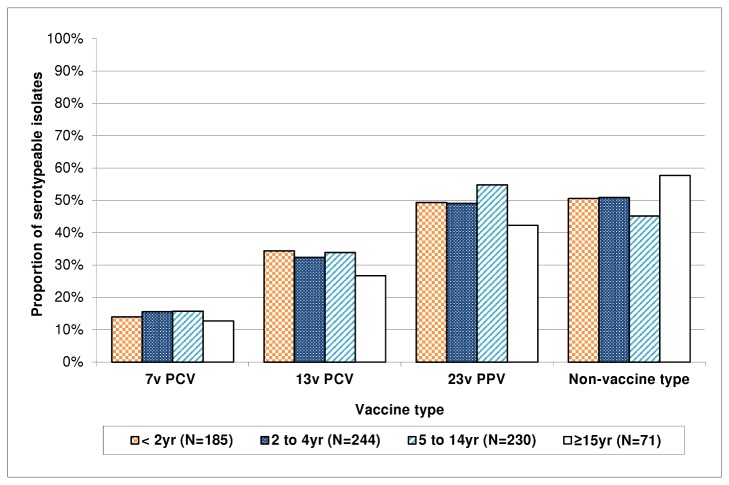
Proportion of serotypeable pneumococci isolated from the nasopharynx between 2008 and 2011 that are included in 7vPCV, 13vPCV, 23vPPV, or no currently licensed vaccine, by age group. A 7vPCV program was introduced in 2001 for all Aboriginal children. Specimens were collected prior to introduction of 13vPCV in July 2011.

Overall, we found 14.5% of serotypeable pneumococci were 7vPCV serotypes, equivalent to a prevalence of 7.0%. 13vPCV (which covers four of the 12 most common serotypes found in this study, namely 6A, 19A, 19F and 23F, but not 6C) would have covered 32.4% of serotypes carried during the study period across all ages. Half (50.1%) of the serotypes found are not included in any currently licensed vaccine (Figure 3). The proportion of serotypes covered by each of the three vaccines varied little with age, although a higher proportion of pneumococci were non-vaccine serotypes in adults than in younger people (Figure 3).

### Antimicrobial susceptibility

Resistance to cotrimoxazole was present in 263 (30.6%) of all 857 pneumococcal isolates, while 156 (18.2%), 82 (9.5%), 14 (1.6%), one (0.001%) and one (0.001%) isolates were resistant to erythromycin, tetracycline, penicillin (MIC ≥ 2µg/ml), chloramphenicol and ceftriaxone (MIC ≥ 2µg/ml), respectively (Table 2). Reduced susceptibility to penicillin (MIC ≥ 0.125µg/ml) was present in 227 (26.5%) isolates. Of the 58 serotype 19A isolates, 28 (48.3%) were resistant to cotrimoxazole and 27 (46.6%) had reduced susceptibility to penicillin; 38 (97.6%) of 41 19F isolates had reduced penicillin susceptibility. Complete penicillin resistance (MIC ≥ 2µg/ml) was identified in nine isolates (1.2%): three (5.2%) serotype 19A, two (4.7%) 19F and one (7.1%) 22A isolate. Chloramphenicol resistance was found in a single isolate of serotype 15C. Multi-resistance to tetracycline, cotrimoxazole and erythromycin was observed in 36 (83.7%) serotype 23F isolates, and seven (5.5%) non-serotypeable isolates. Of 126 non-serotypeable isolates, 59 (46.9%) were resistant to cotrimoxazole and 101 (80.2%) had reduced penicillin susceptibility, five of which were completely resistant to penicillin.

## Discussion

This is the first assessment of pneumococcal carriage in Aboriginal people in WA in the PCV era. The PCV program aimed to reduce the burden of invasive pneumococcal disease (IPD) but we also anticipated that it would alter the serotypes that are carried in the nasopharynx. While pneumococcal carriage rates were high, 7vPCV serotypes were rarely carried. Despite a lack of data on carriage serotypes in WA prior to introduction of 7vPCV, it is likely that the vaccination program has contributed to reducing carriage of 7vPCV serotypes. IPD due to 7vPCV serotypes accounted for 40% of IPD in 1997-2001 compared with 12% in 2005-2007 [2]. The high carriage rates of non-7vPCV serotypes may counterbalance the benefits of the vaccine, assuming that some non-7vPCV serotypes may display similar or higher disease potential [14]. 

The high pneumococcal carriage rates found in this study correspond well with studies in the Northern Territory of Australia where prevalence of carriage surpassed 80% in children aged 2-4 years in a cross-sectional study carried out in 2002 and 2004 [15]. Pneumococcal carriage rates were lower in older adults in WA than Mackenzie et al. found in the Northern Territory but too few samples were collected from people aged ≥ 65 years in our study to enable accurate comparison. Elsewhere in the world, studies in indigenous populations and in developing countries have found comparably high rates of pneumococcal carriage [16–18]. Risk factors for high carriage rates in Australian Aboriginal people including crowding and indoor smoking [4] were widespread among our study participants.

Four of the 12 most common carriage serotypes (6C, 16F, 23B and 34) are not covered by any currently licensed vaccine (Figure 3). Serotype 6C was the most frequently carried serotype in our study (Table 2). In Alaska and the UK, carriage of serotype 6A declined following 7vPCV programs, while the proportion of 6C carriage isolates increased [7,19]. While data on carriage of 6A in WA are not available for the pre-7vPCV era, it is possible that a similar “replacement” took place following the 7vPCV program, given that 6C was the most commonly carried serotype in our study. Vaccination with 7vPCV, which includes 6B, elicits cross-reactive antibodies to 6A but does not appear to protect against disease caused by 6C [20,21]. Meanwhile Cooper et al. have reported that 13vPCV elicits cross-protective functional antibodies to 6C in addition to covering 6A and 6B [22]. We could expect that carriage of serotype 6C may now decrease following the introduction of the 13vPCV program for all children. 

Serotype 16F has been one of the most common carriage serotypes across Australia since the introduction of 7vPCV and is a predominant cause of OM and tympanic membrane perforation in Australian Aboriginal children [23]. Serotype 19A was the third most common serotype isolated in our study, and was the most common serotype in children < 2 years of age. We expect to see a reduction in carriage of 19A over the coming years following the introduction of 13vPCV. Despite their inclusion in 7vPCV, serotypes 23F and 19F were the fourth and fifth most prevalent serotypes found in this study. This was unexpected and requires monitoring over the coming years to determine whether the 13vPCV program reduces carriage of these serotypes. The high frequency of nonserotypeable isolates circulating in the population warrants close monitoring, and improved techniques are needed to identify whether these are non-capsular strains or novel serotypes. 

 23vPPV serotypes made up half (49.9%) of all serotypes found in this study. 23vPPV was included on the immunization schedule for Aboriginal children at 18 months of age and for Aboriginal adults ≥ 55 years in WA. The relatively high prevalence of 23vPPV serotypes in this study suggests it has little effect on carriage. Outbreaks of IPD caused by serotypes 12F and 19A in 2010 and 2011 indicate 23vPPV may not have been effective in preventing IPD or this may reflect the limited 23vPPV coverage in WA [2,24]. We rarely identified serotypes 1 and 12F in the nasopharynx (Table 2), despite the outbreaks of IPD due to these serotypes during the study period [5]. It is not possible to determine whether we were observing replacement disease due to “vaccine pressure” or whether this was part of the natural variation in incidence of these serotypes. This could be expected due to the opportunistic nature of our carriage surveillance resulting in swabs not being collected at the time and in locations where serotype 1 or 12F was circulating. Furthermore, serotypes 1 and 12F have high invasive potential which suggests more transient carriage, so they are rarely isolated from the nasopharynx even in very large carriage studies [25–27]. 

Between 1999 and 2003 carriage rates were almost 50% lower in non-Aboriginal children than in Aboriginal children in the Kalgoorlie-Boulder cohort [12]. During that time period Aboriginal children began receiving 7vPCV while non-Aboriginal children did not routinely receive 7vPCV until 2005. More recently, between November 2007 and May 2009, a cohort of 186 non-Aboriginal children < 36 months old experiencing recurrent acute OM (rAOM) and 81 healthy controls were recruited in a carriage study in WA. *S. pneumoniae*, non-typeable *H. influenzae* and *M. catarrhalis* were carried by 41%, 56% and 43% of the rAOM children and 26%, 19%, and 15% of controls [28]. The carriage rates in both the rAOM and control groups are considerably lower than we found in Aboriginal children of the same age in this study. Ongoing simultaneous surveillance of carriage in the Aboriginal and non-Aboriginal populations is required to examine and compare the effect of vaccination in both groups and give a broad overview of carriage in the WA population. Surveillance of bacterial nasopharyngeal carriage in non-Aboriginal children < 5 years has recently begun in the metropolitan Perth area, and will enhance our overview of carriage in the region. 

Pneumococcal antibiotic resistance remains relatively uncommon in this population apart from cotrimoxazole, which is important when considering empiric therapy for common infections such as OM and pneumonia. Reduced susceptibility to penicillin was observed in one-quarter of isolates, but was particularly common in nonserotypeable isolates that were also more likely to be resistant to cotrimoxazole and erythromycin. We compared our antibiotic susceptibility data with resistance rates for 261 isolates (Lehmann et al., unpublished data) from a cohort of 100 Aboriginal children born in Kalgoorlie-Boulder and followed for 2 years between April 1999 and January 2003 [29]. Cotrimoxazole resistance was more common in our isolate collection (30.7% versus 22.6%, p = 0.01), but susceptibilities to the other antimicrobial agents were not significantly different. It will be important to continue to monitor antibiotic susceptibility for the emergence of multi-resistant strains. 

Our study had some limitations. The opportunistic nature of swab collection may not give a representative sample of the population. Sampling in adults was biased towards women: 77% of adults aged over 20 years were female because generally mothers and grandmothers accompanied their children to medical services, where we had the opportunity to ask them if they would like to participate in the study. However, carriage rates were not significantly different in men and women aged ≥ 20 years (p = 0.79). Vaccination data from ACIR could only be accessed for 56.4% of children swabbed, so our findings may not reflect the true number of vaccinated children. The large expanse of land and diverse regions covered by our surveillance could give rise to seasonal or geographical variation in prevalence which we could not ascertain as samples were frequently collected from limited numbers of people living in many different communities visited in different years and seasons. 

Since July 2011, 13vPCV has replaced 7vPCV in the WA immunization schedule. We therefore anticipate a shift in serotype distribution, with a decrease in circulation of the six additional 13vPCV serotypes now covered in the immunization schedule. Over 30% of our isolates were 13vPCV serotypes. It is essential to closely monitor changes in carriage and IPD (rates, serotype distribution and antimicrobial susceptibility patterns) over the coming years to ensure appropriate vaccine policies are in place and to achieve the best outcome for the WA Aboriginal population [30]. 
